# Synergistic effects of overweight/obesity and high hemoglobin A1c status on elevated high-sensitivity C-reactive protein in Chinese adults: a cross-sectional study

**DOI:** 10.3389/fnut.2023.1156404

**Published:** 2023-05-05

**Authors:** Qianqian Shen, Tingchao He, Ting Li, Ignatius Man-Yau Szeto, Shuai Mao, Wuxian Zhong, Pin Li, Hua Jiang, Yumei Zhang

**Affiliations:** ^1^Department of Nutrition and Food Hygiene, School of Public Health, Peking University, Beijing, China; ^2^Inner Mongolia Dairy Technology Research Institute Co., Ltd., Hohhot, China; ^3^Yili Maternal and Infant Nutrition Institute, Inner Mongolia Yili Industrial Group Co., Ltd., Hohhot, China; ^4^National Center of Technology Innovation for Dairy, Hohhot, China; ^5^School of Nursing, Peking University, Beijing, China

**Keywords:** interaction, hs-CRP, HbA1c, BMI, Chinese adults

## Abstract

**Background:**

High-sensitivity C-reactive protein (hs-CRP) is an inflammatory marker that has been suggested as a predictor of cardiovascular diseases. High glycated hemoglobin (HbA1c) levels and overweight/obesity are independently associated with elevated hs-CRP; meanwhile, high HbA1c levels are frequently accompanied by overweight or obesity. However, their joint effect on elevated hs-CRP levels has not been well-established. Therefore, we evaluated whether overweight/obesity modified the association between high HbA1c levels and elevated hs-CRP.

**Methods:**

Based on cross-sectional data from the Chinese Urban Adults Diet and Health Study (CUADHS) in 2016, we included 1,630 adults aged 18–75 years (mean age 50.16 years and 33.6% male). Elevated hs-CRP was defined as serum hs-CRP ≥ 3 and <10 mg/L. The interactive effects of BMI and HbA1c levels on the risk of elevated hs-CRP levels were calculated by using multiple logistic regression models, followed by strata-specific analyses.

**Results:**

Individuals with elevated hs-CRP had a higher rate of HbA1c level than those without elevated (25.3 vs. 11.3%, *P* < 0.001), as well as a higher rate of overweight/obesity (67.1 vs. 43.5%, *P* < 0.001). Higher HbA1c levels were independently associated with an increased risk of elevated hs-CRP [adjusted odds ratio (aOR) = 2.31, 95% confidence interval (CI): 1.47, 3.65], as well as overweight/obesity with the risk of elevated hs-CRP (aOR = .31, 95% confidenc–3.73). Furthermore, overweight/obesity showed a significant synergistic effect on high HbA1c levels with a higher aOR of 5.25 (2.77, 9.95) (*P*_*interaction*_ < 0.001). This synergistic effect was more prominent when stratified by age (in 18–44 years old, aOR, 95% CI = 30.90, 4.40–236.47 for interaction vs. 6.46, 1.38–30.23 for high HbA1c only) and gender (in women, aOR, 95% CI = 8.33, 3.80–18.23 for interaction vs. 2.46,1.38–4.40 for high HbA1c only).

**Conclusion:**

There are synergistic effects of high HbA1c levels and overweight/obesity on the risk of elevated hs-CRP in Chinese adults, with more significant effects in adults aged 18–44 years or females. Intervention strategies for preventing high blood glucose levels and body weight simultaneously may be important for reducing hs-CRP-related diseases. Further studies are needed to confirm this finding in other populations, and its molecular mechanisms need to be elucidated.

## Introduction

High-sensitivity C-reactive protein (hs-CRP) is an acute-phase reactant and a biomarker of systemic inflammation. Chronic elevated hs-CRP may have biological effects on endothelial function, coagulation, fibrinolysis, oxidation of low-density lipoproteins (LDL), and stability of atherosclerotic plaque (1). Hs-CRP is considered an optimal predictor for the assessment of future cardiovascular events according to the recommendation of the American Heart Association (AHA) (2). It is well-known that cardiovascular disease (CVD) is the leading cause of death globally, which places a substantial burden on health and economy. A meta-analysis of 22 studies indicated that the concentrations of hs-CRP greater than or equal to 3 mg/L were associated with a 60% increased risk of CVD (3), and other studies (4–6) reached similar conclusions including the Chinese population (7). In addition, elevated hs-CRP levels have also been shown to be a risk factor for other diseases. For example, high hs-CRP was significantly associated with a 40% increased risk of depression in younger adults (8, 9) and a 60% increased risk of fractures in elderly men (10). As a result, there is an urgent need to take measures to prevent and control the elevation of hs-CRP to reduce the risk of related diseases.

Glycated hemoglobin (HbA1c) is the amount of hemoglobin in the blood that binds to glucose, reflecting the average blood glucose level over the last 2–3 months. Diabetes is diagnosed the level of HbA1c is 6.5% or higher, according to current guidelines (11, 12). HbA1c above 7% puts diabetics at risk of developing macrovascular and microvascular complications (13–15). Recently, an increasing number of studies have shown that higher HbA1c levels are associated with a greater risk of cardiovascular-related disorders even in non-diabetic subjects (16–19). Elevated HbA1c level is an established risk factor for cardiovascular (CV) complications (20, 21). Therefore, maintaining HbA1c within the normal range could reduce the risk of cardiovascular events. However, there is little evidence to suggest that could confirm the association between the levels of HbA1c and inflammatory biomarkers of CVD, such as hs-CRP.

High HbA1c status is frequently accompanied by being overweight or obese (22), while some studies have observed a relationship between high HbA1c status and higher hs-CRP levels, it is still unclear whether this relationship exists after adjusting for adiposity. Obesity is a chronic metabolic condition that is related to a high risk of CVD, hypertension, insulin resistance and other comorbidities. The latest reported data showed that more than 34.3 and 16.4% of Chinese adults (≥18 years) were overweight and obese, respectively; meanwhile the global prevalence of obesity continues to grow slowly and remains at a high level (23, 24). Studies have shown that inflammatory cytokines produced by adipose tissue, such as tumor necrosis factor-α (TNF-α) and interleukin-6 (IL-6), promote the production of hs-CRP in the liver (25, 26). Consequently, there is a strong positive correlation between obesity indicators, such as waist circumference (WC) and body mass index (BMI), and hs-CRP (27, 28).

Several researchers suggested that inflammation may be a link between obesity, type 2 diabetes and CVD. However, as far as we know, little is known about their combined effects of HbA1c level and overweight/obesity on increased risk of hs-CRP, especially in Chinese adults. In this study, we hypothesized that high HbA1c levels and overweight/obesity, both individually and jointly, are associated with elevated hs-CRP in Chinese adults. If this connection can be proven, lifestyle interventions such as the control of body weight and blood sugar levels can play a primary preventive role in the occurrence of CVD-related diseases by reducing the level of hs-CRP.

Therefore, to evaluate this hypothesis, we conducted this study to explore the interaction between overweight/obesity and high HbA1c status on elevated hs-CRP in adults of the Chinese population.

## Methods

### Study design and population

The data used in the research were drawn from the Chinese Urban Adults Diet and Health Study (CUADHS), which was a cross-sectional study that covered eight cities, representing different economic development and public resources regions in China. From March to July 2016, this survey used a multistage sampling method to randomly recruit 1739 urban adults between 18 and 75 years old from Beijing, Guangzhou, Xuchang, Jilin, Wuhan, Lanzhou, Chenzhou and Chengdu [details have been described elsewhere (29)]. Those with mental illness, memory problems, physical disabilities, or women who were pregnant or lactating were excluded. A series of questionnaires, physical examinations, and blood samples were collected on the day of enrollment. This study was conducted by the biomedical ethics committee of Peking University (No. IRB00001052-15059) and obtain the informed written consent of all participants.

In the current analysis, participants were excluded if they had missing data on high-sensitivity C-reactive protein (hs-CRP) (*n* = 16), glycated hemoglobin (HbA1c) (*n* = 28), or body mass index (BMI) (*n* = 5). Moreover, participants with hs-CRP levels equal to or higher than 10 mg/L were excluded from the analysis (*n* = 33), as those values are indicative of current acute infection (30). Accordingly, 1,630 individuals were included in the final analysis.

### Data collection and definition

Data on sociodemographic characteristics, lifestyle behaviors and individual health status were self-reported and recorded by uniformly trained interviewers, including age, gender, race, marital status, education, income, tobacco smoking, alcohol consumption, physical activities and disease history. Physical examination was performed simultaneously. Height and body weight were measured following standard protocols using SECA877, and BMI was calculated as the weight in kilograms divided by height in meters squared. Then, it was divided into two groups (<24 or ≥24 kg/m^2^) based on the Working Group on Obesity in China criteria (24). Using Omron HEM-7124 electronic blood pressure (BP) monitor, BP was measured twice after sitting for 5 min. The criterion for hypertension was average systolic BP ≥140 mmHg and/or average diastolic BP ≥90 mmHg or having a history of hypertension or having taken prescription drugs to treat hypertension.

Blood samples were collected in the morning after overnight fasting to detect serum Hs-CRP and HbA1c. All samples were analyzed by the Lawke Health Laboratory with strict quality control. The concentration of serum hs-CRP was determined by immunoturbidimetry (Roche Diagnostic). When the serum hs-CRP is above 3 mg/L and below 10 mg/L, it is considered that the individual is in a state of chronic inflammation and has a high risk of CVD. For the present study, serum hs-CRP level was stratified into two degrees (<3 or ≥3 mg/L), and hs-CRP ≥3 mg/L was defined as elevated according to the American Heart Association and Centers for Disease Control and Prevention (30). HbA1c was measured with high-performance liquid chromatography (Tosoh, Tokyo, Japan). We defined higher HbA1c levels as HbA1c ≥ 6.5% and lower HbA1c levels as < 6.5% (31). Individual with TC ≥ 6.22 and/or TG ≥ 2.26 mmol/L, and/or HDL-D < 1.04 and/or LDL-D ≥ 4.14 mmol/L or previous diagnosis with dyslipidemia were identified as dyslipidemia (32).

### Statistical analysis

Data were tested for normality before statistical analysis. If continuous variables didn’t follow a normal distribution, median and interquartile ranges were presented and compared using Mann-Whitney U tests; otherwise, mean ± standard deviation (SD) and Student’s *t*-tests were utilized. Categorical variables are presented as frequencies and percentages and were compared using chi-squared tests.

Multivariable logistic regression was performed and odds ratios [OR, with 95% confidence intervals (CI)] were calculated to analyze the individual and joint associations of BMI and HbA1c with elevated hs-CRP levels in this population. In multivariate logistic regressions, model 1 adjusted for age, gender, race, education level, marital status, and monthly household income, and model 2 further adjusted for city grade, hypertension, dyslipidemia, alcohol use, and smoking status. We tested for interaction effects on a multiplicative scale. For multiplicative interaction, we calculated two-sided *P*-values to evaluate the significance of each product term in the logistic regression models and compared the ORs for HbA1c and elevated hs-CRP in different BMI layers (33).

To further clarify the association, we performed stratified analyses according to age and sex to explore potential disparities in the association between the interaction of BMI and HbA1c on hs-CRP in model 2. All analyses were performed in SPSS Statistics version 24. Two-sided *p*-values less than 0.05 were considered significant.

## Results

### Basic characteristics

The demographic characteristics of study participants by elevated hs-CRP are summarized in Table 1. Among the 1,630 participants [547 men (33.6%)], the mean (± SD) age was 50.16 ± 17.33 years, most were of Han nationality (97.4%), 26.0% had a university education or above and 38.1% of the participants were living in first-tier developed cities. The prevalence of elevated hs-CRP was 8.96% (*n* = 146). Individuals with elevated hs-CRP had higher HbA1c levels than those without elevated hs-CRP (25.3 vs. 11.3%, *P* < 0.001), as well as a higher rate of overweight/obesity (67.1 vs. 43.5%, *P* < 0.001). Additionally, compared with participants with normal hs-CRP levels, those with elevated hs-CRP tended to be older, had poorer educational backgrounds and had a higher level of blood pressure (*P* < 0.05).

**TABLE 1:** Basic demographic characteristics of high-sensitivity C-reactive protein (hs-CRP) elevated^1^ and normal^2^ (*N* = 1,630).

Characteristics	Total	Elevated hs-CRP	Normal hs-CRP	*P*-value
*n* (%)	1,630	146 (8.96)	1,484 (91.04)	–
Age, years	52.09 (34.82, 65.86)	63.47 (43.98, 68.75)	51.34 (33.94, 65.45)	**<0.001**
18–44	611 (37.5)	39 (26.7)	572 (38.5)	–
45–64	556 (34.1)	40 (27.4)	516 (34.8)	–
≥65	463 (28.4)	67 (45.9)	396 (26.7)	–
Gender (%)				0.714
Male	547 (33.6)	47 (32.2)	500 (33.7)	–
Female	1083 (66.4)	99 (67.8)	984 (66.3)	–
City grade (%)				0.883
First-tier city^3^	621 (38.1)	54 (37.0)	567 (38.2)	–
Second-tier city^4^	332 (20.4)	32 (21.9)	300 (20.2)	–
Third-tier city^5^	677 (41.5)	60 (41.1)	617 (41.6)	–
Race (%)				0.316
Han Chinese	1587 (97.4)	144 (98.6)	1443 (97.2)	–
Ethnic minorities	43 (2.6)	2 (1.4)	41 (2.8)	–
Educational level (%)				**0.001**
Junior high school and below	530 (32.6)	67 (45.9)	463 (31.3)	–
High school or equivalent	672 (41.4)	51 (34.9)	621 (42.0)	–
University graduate or above	422 (26.0)	28 (19.2)	394 (26.7)	–
Marital status (%)				**0.022**
Unmarried	266 (16.4)	12 (8.3)	254 (17.2)	–
Married	1250 (76.9)	123 (84.8)	1127 (76.1)	–
Divorced or widowed	110 (6.8)	10 (6.9)	100 (6.8)	–
Monthly household income, ¥				0.589
<5,000	815 (50.3)	76 (52.1)	739 (50.1)	–
5,000–9,999	511 (31.5)	48 (32.9)	463 (31.4)	–
≥10,000	295 (18.2)	22 (15.1)	273 (18.5)	–
Smoking status (%)				0.479
Never	1243 (76.8)	118 (80.8)	1125 (76.4)	–
Former	174 (10.7)	13 (8.9)	161 (10.9)	–
Current	202 (12.5)	15 (10.3)	187 (12.7)	–
Alcohol drinking (%)	453 (27.8)	36 (24.7)	417 (28.1)	0.376
Physical activity, mets/week	1386.0 (693.0, 2772.0)	1567.5 (693.0, 3186.0)	1386.0 (693.0, 2754.0)	0.221
Dyslipidemia (%)	256 (15.8)	30 (20.7)	226 (15.3)	0.090
Hypertension (%)	419 (26.2)	57 (40.1)	362 (24.8)	**<0.001**
SBP, mmHg	122.0 (110.0, 136.0)	130.0 (120.0, 145.3)	120.0 (110.0, 135.0)	**<0.001**
DBP, mmHg	78 (70.0, 85.0)	81.5 (73.0, 88.0)	78.0 (70.0, 85.0)	**<0.001**
Metabolic syndrome (%)	506 (31.0)	89 (51.1)	417 (28.6)	**<0.001**
BMI, kg/m^2^ (%)	23.55 (21.37, 26.01)	25.73 (23.20, 28.02)	23.40 (21.23, 25.81)	**<0.001**
<24	887 (54.4)	48 (32.9)	839 (56.5)	–
≥24	743 (45.6)	98 (67.1)	645 (43.5)	–
Fasting blood glucose, mmol/L	5.22 (4.78, 5.76)	5.58 (5.02, 6.35)	5.19 (4.77, 5.70)	**<0.001**
HbA1c (%)	5.80 (5.50, 6.10)	6.10 (5.60, 6.50)	5.70 (5.50, 6.10)	**<0.001**
<6.5	1425 (87.4)	109 (74.7)	1316 (88.7)	–
≥6.5	205 (12.6)	37 (25.3)	168 (11.3)	–

Data are expressed as the median (interquartile range, IQR) for non-normally distributed continuous variables, as the means ± SDs for normally distributed continuous variables or as counts (percentages) for categorical variables. SBP, systolic blood pressure; DBP, diastolic blood pressure; BMI, body mass index.

^1^Elevated hs-CRP group: hs-CRP ≥ 3 mg/L.

^2^Normal hs-CRP group: <3 mg/L.

^3^Beijing, Guangzhou.

^4^Chengdu, Lanzhou.

^5^Xuchang, Jilin, Wuhu, Chenzhou. The bold values mean that the differences are statistically significant.

### Individual associations of BMI and HbA1c with elevated hs-CRP

Table 2 shows the relationship between BMI and HbA1c and the risk of elevated hs-CRP levels in the overall population, respectively. In the binary logistic regression analyses, by comparison to the lower HbA1c levels, the crude OR (95% CI) of elevated hs-CRP in the higher HbA1c group was 2.66 (1.77, 3.99) (*P* < 0.001). When adjusted for age, sex, race, educational level, marital status, monthly household income, city grade, hypertension, dyslipidemia, alcohol use and smoking status, higher HbA1c levels were still significantly associated with an increased risk of elevated hs-CRP [adjusted OR 2.31 (1.47, 3.65), *P*_*adjusted*_ ≤ 0.001]. Similarly, compared to those who were normal/underweight, those overweight/obese had a crude OR (95% CI) of 2.66 (1.85, 3.81) (*P* < 0.001) for elevated hs-CRP. After adjusting for potential confounding factors, a higher BMI level remained significantly associated with the risk of elevated hs-CRP (aOR = 2.51; 95% CI: 1.68–3.73, *P*_*adjusted*_ < 0.001).

**TABLE 2:** Odds ratios [ORs, 95% confidence intervals (CIs)] for the individual associations of body mass index (BMI) and hemoglobin A1c (HbA1c) status with elevated high-sensitivity C-reactive protein (hs-CRP) (*N* = 1,630).

Variables	*n* (%)	Crude OR, 95% CI	*P*-value	Model 1 OR, 95% CI	*P*-value	Model 2 OR, 95% CI	*P*-value
BMI, kg/m^2^							
<24	48 (5.41)	Ref.	–	Ref.	–	Ref.	–
≥24	98 (13.19)	**2.66 (1.85, 3.81)**	**<0.001**	**2.45 (1.67, 3.60)**	**<0.001**	**2.51 (1.68, 3.73)**	**<0.001**
HbA1c, %							
<6.5	109 (7.65)	Ref.	–	Ref.	–	Ref.	–
≥6.5	37 (18.05)	**2.66 (1.77, 3.99)**	**<0.001**	**2.19 (1.41, 3.38)**	**<0.001**	**2.31 (1.47, 3.65)**	**<0.001**

BMI, body mass index. Model 1: age, gender, race, educational level, marital status, monthly household income; Model 2: model 1+ city grade, hypertension, dyslipidemia, alcohol use and smoking status. n (%): numbers and prevalence rates of high high-sensitivity C-reactive protein (hs-CRP) of each layer. The bold values mean that the differences are statistically significant.

### Synergistic interaction of BMI and HbA1c on elevated hs-CRP

Furthermore, to investigate the effect of the coexistence of BMI and HbA1c levels on elevated hs-CRP, participants were divided into four groups based on the levels of BMI and HbA1c: Group 1 (Normal-weight and Normal-HbA1c), Group 2 (Normal-weight and High-HbA1c), Group 3 (Overweight/Obesity-weight and Normal-HbA1c), and Group 4 (Overweight/Obesity-weight and High-HbA1c). Compared with individuals in Group 1, there were significantly increased risks of elevated hs-CRP for individuals in Groups 2, 3, and 4 (*P*_*interaction*_ < 0.001). Compared with Group 1, the adjusted OR (95% CI) for Group 4 was 5.25 (2.77, 9.95) in the entire population, in contrast with 4.07 (1.90, 8.72) for Group 2 and 3.01 (1.90, 4.77) for Group 3. That is, when analyzed jointly, overweight/obesity and high HbA1c levels were synergistically associated with an increased risk of hs-CRP, and the interactive effect was approximately twice as significant as the individual effect. Results are displayed in detail in Table 3.

**TABLE 3:** Odds ratios [ORs, 95% confidence intervals (CIs)] for the interaction associations of body mass index (BMI) and hemoglobin A1c (HbA1c) status with elevated high-sensitivity C-reactive protein (hs-CRP) (*N* = 1,630).

Interaction	*n* (%)	Crude OR, 95% CI	*P*-value	Model 1 OR, 95% CI	*P*-value	Model 2 OR, 95% CI	*P*-value
N-weight* N-HbA1c	36 (4.45)	Ref.	–	Ref.	–	Ref.	–
N-weight* H-HbA1c	12 (15.38)	**3.90 (1.94, 7.86)**	**<0.001**	**3.43 (1.63, 7.24)**	**0.001**	**4.07 (1.90, 8.72)**	**<0.001**
O-weight* N-HbA1c	73 (11.85)	**2.89 (1.91, 4.37)**	**<0.001**	**2.82 (1.81, 4.39)**	**<0.001**	**3.01 (1.90, 4.77)**	**<0.001**
O-weight* H-HbA1c	25 (19.69)	**5.26 (3.03, 9.13)**	**<0.001**	**4.77 (2.62, 8.68)**	**<0.001**	**5.25 (2.77, 9.95)**	**<0.001**

N, normal (<24 kg/m^2^ for BMI or <6.5% for HbA1c); H, high (≥6.5% for HbA1c); O, overweight or obesity (≥24 kg/m^2^ for BMI); Model 1, age, gender, race, educational level, marital status, monthly household income; Model 2, model 1 + city grade, hypertension, dyslipidemia, alcohol use and smoking status; n (%), numbers and prevalence rates of high high-sensitivity C-reactive protein (hs-CRP) of each layer. The bold values mean that the differences are statistically significant.

### Subgroup analyses

**FIGURE 1:**
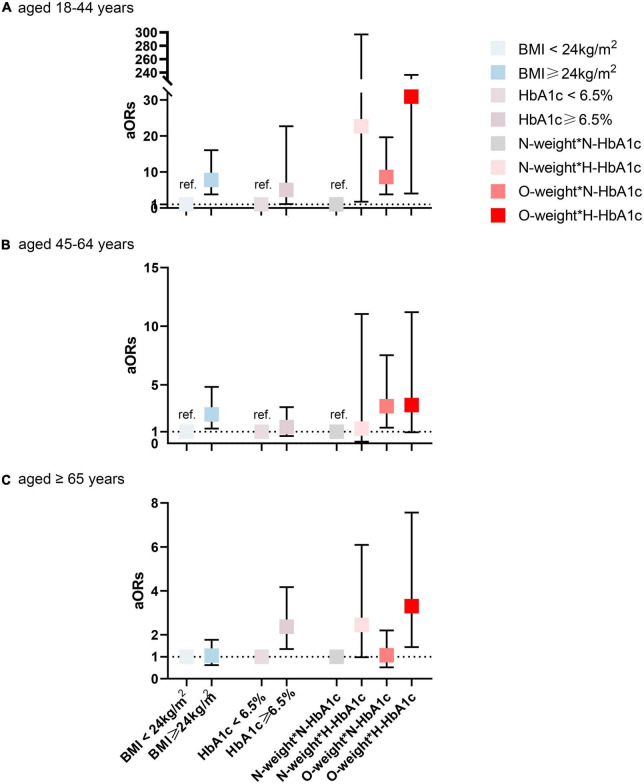
Interaction and independent effect of BMI and hemoglobin A1c (HbA1c) status on elevated high-sensitivity C-reactive protein (hs-CRP), stratified by age. **(A)** aged 18–44 years **(B)** aged 45–64 years **(C)** aged ≥ 65 years. N, normal (<24 kg/m^2^ for BMI or <6.5% for HbA1c); H, high (≥6.5% for HbA1c); O, overweight or obesity (≥24 kg/m^2^ for BMI); BMI, body mass index. Adjusted for gender, race, educational levels, marital status, monthly household income (CNY), city grade, hypertension, dyslipidemia, alcohol use, and smoking status.

**FIGURE 2:**
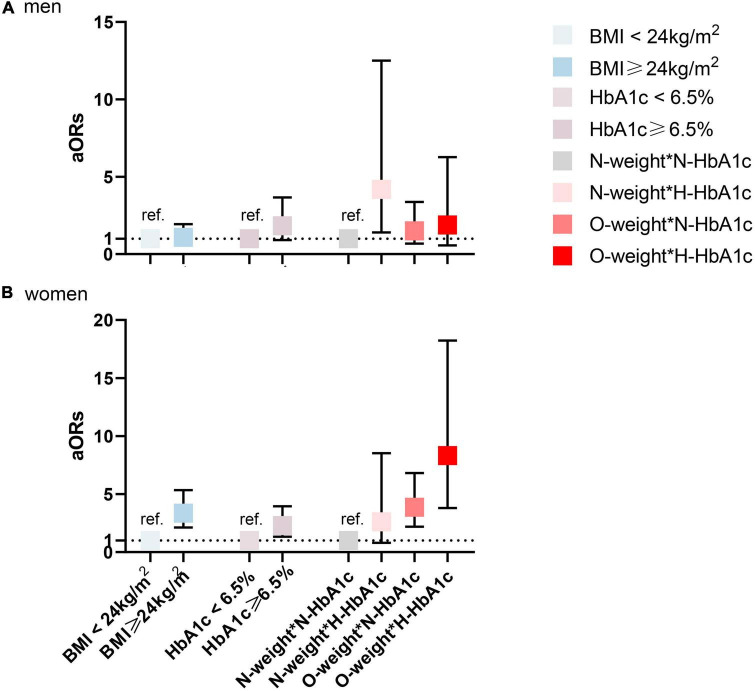
Interaction and independent effect of body mass index (BMI) and hemoglobin A1c (HbA1c) status on elevated high-sensitivity C-reactive protein (hs-CRP), stratified by gender. **(A)** men **(B)** women. N, normal (<24 kg/m^2^ for BMI or <6.5% for HbA1c); H, high (≥6.5% for HbA1c); O, overweight or obesity (≥24 kg/m^2^ for BMI); BMI, body mass index. Adjusted for age, race, educational level, marital status, monthly household income, city grade, hypertension, dyslipidemia, alcohol use, and smoking status.

Next, the above analysis was repeated in two subgroups: age (18–44, 45–64 vs. ≥65 years) and sex (female vs. male). In model 2, the interactions of BMI and HbA1c level with hs-CRP were evaluated to determine whether the joint effect was the same in each subgroup. Stratified analysis by age and gender in model 2 is shown in Figure 1 (age) and Figure 2 (gender). Among those aged 18–44, the interaction effects of overweight/obesity and high HbA1c level on elevated hs-CRP were nearly 6 times more likely than the total population (aOR = 30.90, 95% CI: 4.04–236,47, *P*_*interaction*_ = 0.001) (Supplementary Table 1). It is worth noting that the 95% CI was wide, indicating that the excess risk may be an accidental finding due to the small number of cases in this age range. Interestingly, there were no significant interactions in the population aged 45–64 (aOR = 3.27, 95% CI: 0.95–11.20, *P* = 0.060). Among those over 65 years old, the interaction was weaker (aOR = 3.30, 95% CI: 1.44–7.56, *P* = 0.049). In the stratified analysis by sex, after adjustment for potential confounders, women with high HbA1c levels and overweight/obesity were nearly twice as likely to have elevated hs-CRP than the total population (aOR = 8.33, 95% CI: 3.80–18.23, *P*_*interaction*_ < 0.001) (Supplementary Table 2). However, this association became non-significant in men (aOR = 1.89, 95% CI: 0.57–6.27, *P*_*interaction*_ = 0.301). After stratification by age and sex, the unadjusted and adjusted ORs (95% CIs) according to models 1 and 2 for each layer are attached in the Supplementary material.

## Discussion

In our study of 1,630 Chinese adults, we found that high HbA1c levels and overweight/obesity were independent predictors of hs-CRP elevation, even after considering several traditional confounding factors. Significant interaction effects were observed in the joint analyses, approximately twice as much as the single effect, and the combined effects were stronger in female and younger individuals (aged 18–44 years) for hs-CRP elevation prediction.

Many previous studies have reported a high association between HbA1c and hs-CRP levels. The increase in hs-CRP level is a predictor of a higher risk of cardiovascular events and cardiovascular mortality in the future. A recent study confirmed that HbA1c was favorable for predicting arterial stiffness in the general Chinese population, regardless of whether or not it was in those with a normal glucose status (34). A previous study demonstrated that the serum hs-CRP level was high in patients with diabetes, and the only statistically significant determinants of hs-CRP in this study were gender, BMI, WHR, and concurrent HbA1c (35). Similar to these findings, Wei et al. (36) found that the level of HbA1c was positively correlated with the level of hs-CRP in patients with diabetes according to Pearson correlation analysis. These results suggested an association between HbA1c levels and inflammatory levels in diabetic patients. Meanwhile, in 3,537 Korean adults not diagnosed with diabetes (aged 19–80 years), increases in HbA1c correlated with hs-CRP levels (β = 0.185, *p* = 0.001, and R^2^ = 0.087). In this study, after adjusting for confounders, there was a correlation between fasting glucose and hs-CRP levels in females. However, similar to our research, there was no statistical significance in males (37). Among 1,723 Korean teenagers (aged 10–18 years), in multiple regression analysis, HbA1c levels were significantly associated with hs-CRP (β = 0.036, *p* = 0.012) (38). However, we note that some other studies had contrary results. One study with older adults aged 65–95 in Portugal found that HbA1c was not associated with hs-CRP (39). However, only 118 older adults were enrolled in this study.

Previous studies have demonstrated that higher BMI levels were associated with higher CRP levels, which is consistent with our findings. In a cross-sectional study reported in Chinese adults, after adjusting for confounders, the risk of elevated hs-CRP in overweight group was 1.27 times greater than in normal weight group, and that in the obese group was 1.70 times greater than in normal body weight group (40). Khoo et al. (41) found that BMI was directly related to hs-CRP in all evaluated ethnic groups. The increase in hs-CRP associated with each unit increase in BMI was greater in the Chinese population than in other ethnic groups. Another study suggested that Asians had higher body fat content and a higher risk of diabetes, hypertension, and heart disease than people with the same BMI of other races (42). In other races and populations, the results are similar. Among middle-aged and olderly African-Americans enrolled in the Jackson Heart Study, hs-CRP was used as a measure of inflammation, and there was a strong correlation between BMI and hs-CRP. In a case–control study conducted in Australia, MacKenzie et al. (43) found that hs-CRP was related to BMI z- score (r = 0.47, *p* < 0.001) in children with type 1 diabetes mellitus (T1DM). In Cuban Americans, Huffman et al. (44) showed that BMI was significantly associated with ln hs-CRP whether with type 2 diabetes or not. The slope for the relationship between BMI and ln hs-CRP was stronger in people without diabetes was greater than that in people with diabetes (β = 0.099 and β = 0.055, respectively), but both slopes were significantly different than zero (*p* < 0.001). That is, there is an interaction between BMI and diabetes status on the risk of elevated hs-CRP in this population. This study seems to show that their relationship is stronger in women than in men, which is similar to our findings.

To our knowledge, this is the first study to examine the interactions between HbA1c levels, BMI and their interaction on the risk of hs-CRP elevation among adults in China. Therefore, the current analysis is largely exploratory in nature but serves to add clinical insight into the inflammatory burden of Asian populations.

Obesity induces adipocyte dysfunction, with adipokine secretion and macrophage activation leading to proinflammatory cytokine such as tumor necrosis factor alpha (TNF-α) and interleukin-6 (IL-6) production and the release of anti-inflammatory adipokines such as adiponectin reduction (45). Inflammatory adipocytokines TNF-α and IL-6 are associated with elevated levels of circulating hs-CRP (26). These changes in the release of adipocytokines, especially from visceral adipose tissue, induce a state of systemic insulin resistance and low-grade inflammation (45). Being overweight and obese is known to be associated with insulin resistance and increased glycemia. Previous studies have explored the association between serum glucose and inflammation. Some studies have suggested that reactive oxidative species from glycation end products are a pro-inflammatory effect of increased glucose levels (46). Another plausible mechanism is that hyperglycemia affects NF-κb, a key mediator that regulates multiple proinflammatory and proatherosclerotic target genes in endothelial cells, vascular smooth muscle cells, and macrophages (47). When the overweight/obesity and high HbA1c levels exist at the same time, they have a synergistic effect on the increase in hs-CRP.

Our investigation also revealed the effect of their interaction on hs-CRP after stratification by sex and age. Our results show that the interaction between overweight/obesity and high HbA1c levels was significant in females. However, this effect was not observed in males. Our findings are similar to those of Nari et al. (48). Two mechanisms have been suggested. First, estrogen secretion in female individuals may play a role in the etiology of inflammation (49), resulting in a more pronounced effect on hs-CRP levels. Second, females generally have higher levels of the body and visceral fat than males (50), with a consequent increase in hs-CRP levels and CVD risk. Moreover, this joint effect was not attenuated in the age-specific analysis and was observed in both younger and older individuals. Interestingly, this effect was not observed in middle-aged people. In future research, the sample size can be expanded for further analysis.

Overall, our study has important clinical and public health implications. The gate for the prevention and control of CVD-related diseases moved forward from controlling the occurrence of CVD to controlling the level of hs-CRP. It is clear from our study that reducing HbA1c levels and controlling body weight are important for reducing the risk of hs-CRP-related diseases in the future. Rossello et al. (16) clarified the clinical significance of routine detection of HbA1c levels in non-diabetic subjects, which can not only prevent the development of diabetes, but also estimate the risk of subclinical atherosclerosis (SA) and subsequent CVD, and monitor the effectiveness of therapeutic interventions to address this risk. In routine clinical practice, measurement of HbA1c levels is not always performed in subjects without diabetes, while levels below the diabetic range are also often left untreated. Our study provides a scientific basis for paying attention to and controlling HbA1c levels and body weight in non-diabetic patients to help reduce inflammation. For individuals, simultaneous control of body weight and blood sugar levels can reduce inflammation better than a single indicator, especially for women and people aged between 18 and 44 years old. Individuals with excess body weight or high HbA1c status are believed to benefit from early diet and lifestyle intervention. For health professionals, to improve the early identification of CVD-related diseases, more attention should be given to the body weight and HbA1c level of this population simultaneously in clinical practice, which may have greater clinical significance for the prevention and treatment of these diseases.

The primary strength of this study was that our study quantitatively shows for the first time the effects of overweight/obesity and high levels of HbA1c on inflammation, and provides scientific evidence for the prevention and treatment of related diseases caused by hs-CRP. Second, stratified analysis by age and sex was performed to clarify different effects between the combined BMI and HbA1c and elevated hs-CRP levels. Third, this study was carried out among the general population in eight cities in China, and multistage stratified cluster sampling was conducted, which is a nationally representative survey, improving the representativeness of the findings for the Chinese population.

The current study nevertheless had several limitations. First, as this was a cross-sectional study, we could not infer causality and temporality relationship between overweight/obesity, high HbA1c levels and elevated hs-CRP, and there might be recall bias. Second, the sample size of this study is relatively small, and the extrapolation of the research results is affected to a certain extent. Third, despite extensive adjustment for potential confounders, residual confounding factors cannot be completely ruled out, such as a history of CVD-related disease and a family history of the disease. Finally, this study was conducted in the general population of China, without distinguishing whether diabetes was present, and the results may be easily affected by diabetes status. In the future, separate studies can be conducted specifically in diabetic and non-diabetic people to better determine whether there is interaction and the size of the effect in different diabetic states.

## Conclusion

In conclusion, high HbA1c levels and overweight/obesity, both individually and jointly, were associated with elevated hs-CRP risks in Chinese adults, especially in female and younger individuals. Hs-CRP-related disease prevention strategies aimed at reducing HbA1c levels and reducing body weight simultaneously may exceed the expected benefits based on targeting either risk factor alone. Further studies are required to expand the sample size and validate these findings in different populations. The potential mechanism underlying the joint effect of high HbA1c levels and overweight/obesity needs to be clarified.

## Data availability statement

The original contributions presented in this study are included in the article/Supplementary material, further inquiries can be directed to the corresponding authors.

## Ethics statement

The studies involving human participants were reviewed and approved by the Biomedical Ethics Committee of Peking University. The patients/participants provided their written informed consent to participate in this study.

## Author contributions

QS and YZ contributed to the design of this study. SM, WZ, and PL contributed to the data collation of the manuscript. TH, TL, and IS were responsible for quality control. QS analyzed the data and drafted the manuscript. HJ and YZ contributed to revising this manuscript. All authors read and approved the final manuscript.
