# Supplementary material for: Synergistic effects of overweight/obesity and high hemoglobin A1c status on elevated high-sensitivity C-reactive protein in Chinese adults: a cross-sectional study
Source: Front Nutr. 2023 May 5;10:1156404. doi: 10.3389/fnut.2023.1156404 (PMC10196946; doi:10.3389/fnut.2023.1156404)
Supplement: Supplementary file 1 [file Table_1.docx]

Supplementary Material

Synergistic effects of overweight/obesity and high hemoglobin A1c status on elevated high-sensitivity C-reactive protein in Chinese adults: a cross-sectional study

Qianqian Shen^1^, Tingchao He^2, 3^, Ting Li^2, 3^, Ignatius Man-Yau Szeto^2, 4^, Shuai Mao^1^, Wuxian Zhong^1^, Pin Li^1^, Hua Jiang^5^ *, Yumei Zhang^1^ *

^1^ Department of Nutrition and Food Hygiene, School of Public Health, Peking University, Beijing 100191, China

^2^ Inner Mongolia Dairy Technology Research Institute Co., Ltd., Hohhot 010110, China

^3^ Yili Maternal and Infant Nutrition Institute, Inner Mongolia Yili Industrial Group Co., Ltd., Hohhot, China

^4^ National Center of Technology Innovation for Dairy, Hohhot 010110, China

^5^ School of Nursing, Peking University, Beijing 100091, China

*** Correspondence: These authors contributed equally to this work and share corresponding author**

Corresponding Author:

Yumei Zhang, MD, PhD, E-mail: [zhangyumei@bjmu.edu.cn](mailto:zhangyumei@bjmu.edu.cn);

Hua Jiang, MD, PhD, [jianghua@bjmu.edu.cn](mailto:jianghua@bjmu.edu.cn).

# Supplementary Tables

**Supplement Table S1-1.** Odds ratios [ORs, 95% confidence intervals (CIs)] for the individual associations of BMI and HbA1c status with elevated hs-CRP, stratified by age (N = 1630)

| Variables | **18-44 years** | |  | **45-64 years** | |  | **≥65 years** | |
| --- | --- | --- | --- | --- | --- | --- | --- | --- |
|  | OR, 95%CI | *P* value |  | OR, 95%CI | *P* value |  | OR, 95%CI | *P* value |
| **BMI, kg/m^2^** |  |  |  |  |  |  |  |  |
| <24 | Ref. | - |  | Ref. | - |  | Ref. | - |
| ≥24 | **7.84(3.55, 17.35)** | **＜0.001** |  | **3.11(1.38, 7.01)** | **0.006** |  | 1.17(0.67, 2.05) | 0.587 |
| **HbA1c, %** |  |  |  |  |  |  |  |  |
| <6.5 | Ref. | - |  | Ref. | - |  | Ref. | - |
| ≥6.5 | **6.46(1.38, 30.23)** | **0.018** |  | 1.23(0.48, 3.20) | 0.666 |  | **2.79(1.53, 5.06)** | **0.001** |

BMI: body mass index

Adjusted for gender, race, educational level, marital status, monthly household income, city grade, hypertension, dyslipidemia, alcohol use and smoking status

**Supplement Table S1-2.** Odds ratios [ORs, 95% confidence intervals (CIs)] for the individual associations of BMI and HbA1c status with elevated hs-CRP, stratified by gender (N = 1630)

| Variables | **male** | |  | **female** | |
| --- | --- | --- | --- | --- | --- |
|  | OR, 95%CI | *P* value |  | OR, 95%CI | *P* value |
| **BMI, kg/m^2^** |  |  |  |  |  |
| <24 | Ref. | - |  | Ref. | - |
| ≥24 | 1.05(0.54, 2.03) | 0.887 |  | **3.85(2.31, 6.42)** | **＜0.001** |
| **HbA1c, %** |  |  |  |  |  |
| <6.5 | Ref. | - |  | Ref. | - |
| ≥6.5 | **2.21(1.04, 4.71)** | **0.040** |  | **2.46(1.38, 4.40)** | **0.002** |

BMI: body mass index

Adjusted for age, race, educational level, marital status, monthly household income, city grade, hypertension, dyslipidemia, alcohol use and smoking status

**Supplement Table S2-1.** Odds ratios [ORs, 95% confidence intervals (CIs)] for the interaction associations of BMI and HbA1c status with elevated hs-CRP, stratified by gender (N = 1630)

1. Aged 18-44 years

| Interaction | Crude  OR, 95%CI | *P* value | Model 1  OR, 95%CI | *P* value | Model 2  OR, 95%CI | *P* value |
| --- | --- | --- | --- | --- | --- | --- |
| N-weight*N-HbA1c | Ref. | - | Ref. | - | Ref. | - |
| N-weight*H-HbA1c | **19.36(1.63, 229.83)** | **0.019** | **21.17(1.70, 263.78)** | **0.018** | **22.70(1.74, 297.01)** | **0.017** |
| O-weight*N-HbA1c | **6.97(3.34, 14.52)** | **＜0.001** | **7.66(3.49, 16.80)** | **＜0.001** | **8.59(3.76, 19.62)** | **＜0.001** |
| O-weight*H-HbA1c | **15.49(2.70, 88.76)** | **0.002** | **20.35(3.15, 131.28)** | **0.002** | **30.90(4.04, 236,47)** | **0.001** |

1. Aged 45-64 years

| Interaction | Crude  OR, 95%CI | *P* value | Model 1  OR, 95%CI | *P* value | Model 2  OR, 95%CI | *P* value |
| --- | --- | --- | --- | --- | --- | --- |
| N-weight*N-HbA1c | Ref. | - | Ref. | - | Ref. | - |
| N-weight*H-HbA1c | 0.92(0.11, 7.55) | 0.937 | 1.02(0.12, 8.56) | 0.985 | 1.26(0.14, 11.04) | 0.836 |
| O-weight*N-HbA1c | **2.63(1.20, 5.77)** | **0.016** | **2.59(1.16, 5.78)** | **0.020** | **3.17(1.34, 7.52)** | **0.009** |
| O-weight*H-HbA1c | 2.49(0.80, 7.76) | 0.116 | 2.73(0.86, 8.71) | 0.090 | 3.27(0.95, 11.20) | 0.060 |

1. Aged ≥65 years

| Interaction | Crude  OR, 95%CI | *P* value | Model 1  OR, 95%CI | *P* value | Model 2  OR, 95%CI | *P* value |
| --- | --- | --- | --- | --- | --- | --- |
| N-weight*N-HbA1c | Ref. | - | Ref. | - | Ref. | - |
| N-weight*H-HbA1c | 2.17(0.91, 5.18) | 0.080 | 2.24(0.92, 5.43) | 0.075 | 2.45(0.98, 6.09) | 0.054 |
| O-weight*N-HbA1c | 1.07(0.55, 2.11) | 0.840 | 1.12(0.56, 2.23) | 0.756 | 1.07(0.52, 2.20) | 0.857 |
| O-weight*H-HbA1c | **3.03(1.43, 6.41)** | **0.004** | **3.08(1.44, 6.58)** | **0.004** | **3.30(1.44, 7.56)** | **0.049** |

N: normal (<24 kg/m^2^ for BMI or <6.5% for HbA1c)

H: high (≥6.5% for HbA1c)

O: overweight or obesity (≥24 kg/m^2^ for BMI)

Model 1: gender, race, educational level, marital status, monthly household income.

Model 2: model 1+ city grade, hypertension, dyslipidemia, alcohol use and smoking status

**Supplement Table S2-2.** Odds ratios [ORs, 95% confidence intervals (CIs)] for the interaction associations of BMI and HbA1c status with elevated hs-CRP, stratified by gender (N = 1630)

1. male

| Interaction | Crude  OR, 95%CI | *P* value | Model 1 OR, 95%CI | *P* value | Model 2  OR, 95%CI | *P* value |
| --- | --- | --- | --- | --- | --- | --- |
| N-weight*N-HbA1c | Ref. | - | Ref. | - | Ref. | - |
| N-weight*H-HbA1c | **4.09(1.55, 10.80)** | **0.005** | **3.47(1.21, 9.93)** | **0.020** | **4.19(1.40, 12.51)** | **0.010** |
| O-weight*N-HbA1c | 1.50(0.72, 3.10) | 0.280 | 1.43(0.67, 3.07) | 0.354 | 1.51(0.68, 3.37) | 0.313 |
| O-weight*H-HbA1c | 1.72(0.58, 5.13) | 0.330 | 1.74(0.56, 5.42) | 0.340 | 1.89(0.57, 6.27) | 0.301 |

1. female

| Interaction | Crude  OR, 95%CI | *P* value | Model 1  OR, 95%CI | *P* value | Model 2  OR, 95%CI | *P* value |
| --- | --- | --- | --- | --- | --- | --- |
| N-weight*N-HbA1c | Ref. | - | Ref. | - | Ref. | - |
| N-weight*H-HbA1c | 2.78(0.91, 8.44) | 0.072 | 2.31(0.72, 7.38) | 0.159 | 2.62(0.81, 8.52) | 0.109 |
| O-weight*N-HbA1c | **4.00(2.41, 6.62)** | **＜0.001** | **3.66(2.13, 6.30)** | **＜0.001** | **3.87(2.19, 6.82)** | **＜0.001** |
| O-weight*H-HbA1c | **8.68(4.51, 16.68)** | **＜0.001** | **7.52(3.61, 15.63)** | **＜0.001** | **8.33(3.80, 18.23)** | **＜0.001** |

N: normal (<24 kg/m^2^ for BMI or <6.5% for HbA1c)

H: high (≥6.5% for HbA1c)

O: overweight or obesity (≥24 kg/m^2^ for BMI)

Model 1: age, race, educational level, marital status, monthly household income.

Model 2: model 1+ city grade, hypertension, dyslipidemia, alcohol use and smoking status
